# Relationships between community public service facilities and social capital: An exploratory study of Harbin, China

**DOI:** 10.1371/journal.pone.0318144

**Published:** 2025-02-28

**Authors:** Yichen Luo, Guangtian Zou, Qianyu Liu

**Affiliations:** School of Architecture and Design, Harbin Institute of Technology, Key Laboratory of Cold Region Urban and Rural Human Settlement Environment Science and Technology, Ministry of Industry and Information Technology, Harbin, China; Zhejiang A&F University, CHINA

## Abstract

Social capital is closely intertwined with neighborhood environments and facility provisions. It is a vital resource for supporting residents’ happiness and satisfaction. In the context of urban stock development and the concentrated construction of community public service facilities, it remains unclear whether the spontaneous construction of facilities follows a colocation model and how facility-related construction affects residents’ quality of life. In this study, we used a questionnaire survey to analyze social capital, summarizing it into aspects such as community cohesion, social support, community trust, and sense of belonging. Then, we acquired the points of interest for facilities in Harbin, China, computed the Global Colocation Quotient (GCLQ) and Local Colocation Quotient (LCLQ), the proportion of spatial units occupied by facilities, and the Shannon diversity index, and analyzed the patterns. Aged care services, childcare centers, and community cultural services were found to have the strongest mutual attractions. Finally, an ordinary least squares model was constructed. The proportion of spatial units occupied by community public service facilities was closely related to social capital growth. In the relationship between the colocation model and social capital, LCLQ-healthcare showed a negative correlation, LCLQ-aged care and LCLQ-childcare showed a positive correlation, and LCLQ-cultural and sports showed a positive correlation with a sense of community belonging. The geographically weighted regression model indicated significant spatial heterogeneity in how facility-related construction affects social capital. This study offers a foundation and reference for the sustainable planning and development of integrated community public service facilities.

## 1. Introduction

Social capital arises from social networks, mutual trust, and shared norms that connect individuals [1]. In the context of complete community construction and planning, fostering community social capital is both a key objective and a fundamental assurance of residents’ lives. The construction of community public service facilities can not only meet residents’ daily needs and improve the quality of the neighborhood environment but also provide platforms and opportunities for group interaction, thereby enhancing community cohesion and social capital [2,3]. Guided by policies aimed at promoting and enhancing the functions of comprehensive community service facilities, their construction should follow the principles of efficiency and centralized development [4]. When community public service facilities are independently constructed and operated, they are influenced by residents’ activities and the urban environment and often display clustered distribution characteristics [5]. Under such development trends, the spatial distribution characteristics and intrinsic connections of various facilities, as well as their impact on community social capital, have become critical issues to address in building complete communities.

Analysis of urban facility agglomeration patterns can support studies on urban layouts and public behavioral psychology [6]. The diversification of community public service facilities positively impacts social capital. For example, the establishment of community recreational facilities, sports centers, and senior activity venues promotes community interaction and enhances neighborhood trust [7]. Multifunctional composite facilities, such as those integrating medical care and elder support or promoting intergenerational assistance, provide emotional support and care guarantees [8]. However, increasing neighborhood density and certain commercial facilities hinder the growth of social capital [9]. Nonetheless, some research gaps persist in this field: social capital is significantly influenced by regional cultures [10], measurement scales remain inconsistent, and the impact of composite construction trends for public service facilities on social capital remains unclear.

The symbiotic, connected topological relationships and spatial associations inherent in urban facility construction cannot be overlooked. The density of facility types in spatial units [11], diversity, spatial autocorrelation, and co-location quotient [12] reflect clustering characteristics and patterns in the data. These indicators are mainly used in urban design to study co-location relationships in service industries and catering, associations between urban employment and residential factors, and enterprise location decisions. However, their application to community public service facilities remains limited, with relevant patterns still requiring further exploration. GCLQ effectively identifies the spatial association characteristics of urban facilities [13]. By integrating geographically weighted parameters from spatial regression models into LCLQ, results become less affected by zonal effects, facilitating the investigation of intra-regional patterns.

The above research highlights the necessity of enhancing community social capital through resource integration. This study investigates the influence of the configuration characteristics and colocation patterns of community public service facilities on social capital through three key questions. First, what are the quantified dimensions of social capital after regional adjustments? Second, what are the configuration characteristics and colocation characteristics of six categories of community public service facilities? Third, how do the configuration characteristics and colocation patterns of community public service facilities affect social capital at both global and local levels?

This study explored the configuration characteristics and colocation patterns of six types of community public service facilities, analyzing their impact and extent on social capital, with the aim of investigating the optimization direction of joint construction models of community facilities. Social capital, combined with care, responsiveness, political progress, and resilience, forms the dimensions of social design [14]. As the foundation for achieving collective goals and establishing local communities, social capital concretely responds to Papanek’s concept of designing for the real world [15,16]. Using a social capital perspective, this study provides a reference and basis for the current composite construction of community public service facilities. Regarding long-term development, it provides a data foundation and establishes a direction for planning community public service composite facilities under the concept of social design.

The rest of this paper is structured as follows: Section 2 summarizes the research literature and existing gaps. Section 3 establishes the research framework and describes the methods for data collection and processing based on the research questions. Section 4 analyzes and interprets the findings, followed by a discussion in Section 5. Finally, Section 6 concludes the paper.

## 2. Literature review

### 2.1. Social capital

In defining social capital, Pierre Bourdieu [17] was the first to define it as the sum of actual or potential resources obtained through institutionalized relationship networks. Coleman [18] emphasized that social capital fosters close ties, obligations, and identity among individuals. Robert D. Putnam [19] provided the most widely accepted definition: social capital is the sum of social networks, social trust, and cooperative and reciprocal relationships formed within communities, which fosters collaboration, development, and democratic participation. Social capital is widely applied in research fields such as economic development, health, crime, education, and governance [20].

Communities are regarded as dispersed networks of interpersonal relationships, and social capital can also be defined as localized social capital. It reflects the strength of bonds, trust, and reciprocity among residents [21]. In terms of social capital quantification, tools such as SCAT (Social Capital Assessment Tools) [22] and A-CAT scales (Harpham, Kawachi) have gradually been adopted. The main dimensions include participation in local groups or organizations, localized social networks, informal social interactions, trust, reciprocity, volunteerism, social support, community cohesion, and community sentiment [23].

Overall, while social capital has a profound impact on areas such as community renewal [20] it still lacks a clear definition and systematic quantification. Evaluation scales lack standardized criteria and comprehensive dimensions [24], which constrains validity analysis to some extent [25]. Member relationships within social capital are influenced by historical and cultural contexts [10]. Therefore, the systematic construction of social capital scales needs to account for the critical factor of regional differences.

### 2.2. Relationship between social capital and the construction of community public service facilities

The essence of social organizations’ actions is to achieve trzhust and cooperation within the organization [26]. Social capital, an essential element for enhancing community quality and sustainable community development [27], is inseparable from community construction.

Regarding the dimensions of social capital, the disaster resilience of community public service facilities can significantly enhance community cohesion [28], and the suitability of the neighborhood environment is closely linked to residential satisfaction, a sense of belonging [29], and residents’ place identity [30]. Social capital, urban resource equity, neighborhood sense of place, and community sense are key themes in social design. Existing studies indicate that the construction of community public service facilities is related to these themes [11,31].

In terms of facility function categories, community parks or sports activity centers are conducive to creating communities with positive social capital [32]. Building older adult-friendly communities is closely related to community cohesion [33], community green spaces and landscapes are linked to social support and residents’ health [34], and community entertainment facilities are associated with community interactions [35]. However, an increase in the number of certain commercial facilities can affect neighborhood safety and lead to a decrease in social capital [36]. In the context of facility configuration and layout, macroscale factors, such as land-use intensity, facility construction density, and walkability, are all associated with social capital. The higher the accessibility of facilities, such as clinics, hospitals, community centers, and schools, the more beneficial it is to increase social capital [35]. However, high-density residential environments clearly have detrimental effects on residents’ community interactions [9]. Few scholars have explored how multiple associated amenities working together affect social capital at the meso level, and there is a relative paucity of research on how agglomeration patterns formed by multiple types of amenities within the same community’s living sphere act on social capital.

Existing research methods primarily use structural equation models (SEM) [37], partial least squares structural equation models (PLS-SEM) [38], or ANN neural networks [39] to quantify social capital and related concepts, discussing correlations on a global scale. Urban planning and development inherently feature regional variations (citation), with distinct focal points and functional divisions, a factor insufficiently addressed in existing research. Geographically Weighted Regression (GWR) is commonly applied in accessibility research, offering localized models by fitting regression equations for individual variables, thus overcoming the inability of traditional regression models to reflect spatial variations in coefficients [40]. In practical applications, GWR requires testing with Ordinary Least Squares (OLS) regression models to exclude multicollinearity before conducting further analysis. Therefore, exploring the relationship between social capital and community public service facilities through GWR can take into account the development differences of urban areas, addressing the third research question.

### 2.3. Configuration characteristics and colocation patterns of community public service facilities

The concept of “industry co-agglomeration analysis” was first proposed by Ellison and Glaeser, suggesting that differentiated industries with certain relational ties tend to be spatially proximate [41]. The self-organized spatial layout characteristics of community public service facilities reflect their intrinsic associations and preferences and possess considerable research value in urban planning, geography, and economics.

In quantifying facility configuration characteristics, the proportion of facilities within a unit area reflects the extent to which a region is represented by certain feature types [11], making it more accurate than the area proportion method at smaller spatial scales. The Shannon-Weiner diversity index was proposed in 1948 by Claude Elwood Shannon, the founder of information theory [42]. It is used to measure species richness and the evenness of individual distributions within a system, and is widely applied in fields such as mathematics, communication, and ecology. Compared to balance indices and entropy indices, it offers advantages such as appropriate scalability and no restrictions on sample evaluation, making it a suitable measurement method [43]. In recent years, the research methods of “spatial association” have developed swiftly and refer to patterns of association within a single element or between two or more elements. The concept of colocation patterns was initially proposed by Shekhar and Huang [44], while Leslie and Kronenfeld introduced the Global Co-location Quotients (GCLQ), which enable the detection of spatial associations independent of feature distribution patterns and assess asymmetry in the distribution of spatial elements [45]. Building on this, Cromley introduced the Local Indicator of the Co-location Quotient (LCLQ), which reveals spatially localized variations in the correlation between two sets [46], addressing the GCLQ algorithm’s limitation in overlooking the spatial heterogeneity of settlement patterns. Previous studies have combined two methods to identify regional aging patterns, using GCLQ to determine overall probabilities and LCLQ to reveal specific spatial variations [47]. This approach is applicable for investigating both the overall relationships and the spatial characteristics of community public service facilities.

The layout characteristics of community public service facilities are primarily studied by analyzing industrial spatial distribution patterns via Points of Interest [48], the colocation relationships of industrial clusters [49], and guidelines for medical facility construction [50]. In practical applications, these studies assist in business location decisions [51], identify high-crime regions [52], and pinpoint fire-risk areas [53]. However, research on the overall and regionalized patterns and characteristics of the associative configuration of community public service facilities remains insufficient.

A review of existing research reveals the following:

1. In response to the inconsistent and regionally lacking nature of social capital scales, these scales and indicators need to be refined and supplemented to better meet evolving social needs and lay a solid foundation for future research.
2. Community public service facilities, as a significant part of the neighborhood environment, have unclear characteristics and patterns in their spontaneous construction. The lack of research on the spontaneous construction rules of these facilities within the context of stock development hinders the scientific composite construction of various types of facilities.
3. There is a lack of research on the specific dimensions and the nature of the impact that the associative construction model of facilities has on social capital. Furthermore, there is a shortage of research on this association from the perspective of regional differentiation in urban development.

## 3. Methodology

The participants were informed of the study objectives before the experiment and provided written informed consent. This study was approved by the Harbin Institute of Technology Medical Ethics Committee. For minor participating in the study, consent was obtained from their guardians.

### 3.1. Research frameworks

**Fig 1:**
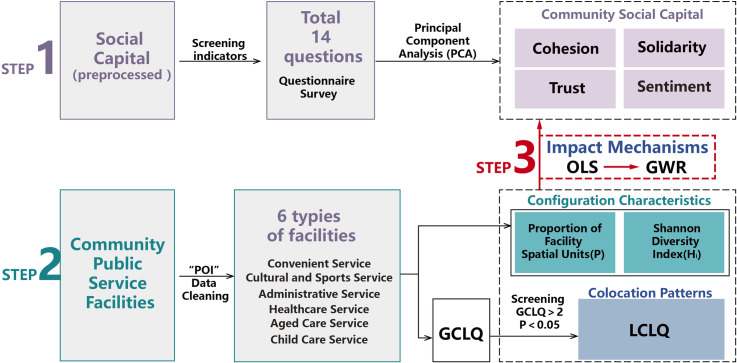
A research roadmap of the impact path of community public service facilities on social capital.

This study calculated the stock of community social capital in residents’ lives through surveys, examining the characteristics of community public service facility construction and its impact mechanisms on social capital across two dimensions: supply the configuration characteristics and colocation patterns of the six types of facilities. The study was divided into three steps. First, residents of the main urban area of Harbin were surveyed using a questionnaire comprising 14 questions related to social capital. The data were quantified and subjected to principal component analysis to summarize the four descriptive dimensions of community social capital. Second, using big data statistics and GIS calculations, the configuration characteristics and colocation patterns of community public service facilities at the sample points were obtained. Configuration characteristics were expressed by the proportion of facility spatial units and the Shannon diversity index. The colocation patterns of facility construction were identified using the global (GCLQ) and local (LCLQ) synergy location entropy values, representing the correlation patterns and degrees. Third, by constructing an OLS multiple regression model, this study explored the impact of community public service facility construction on residents’ social capital. Additionally, a GWR model was used to clarify the spatial heterogeneity of the impact mechanisms within the study area (Fig 1), addressing the limitations of traditional regression models in reflecting local variations in the regression coefficients.

### 3.2. Spatial quantitative analysis methods

(1) Indicators of facility configuration characteristics

In this study, the Shannon-Wiener Diversity Index and the proportion within unit space can reflect the spatial concentration and type diversity of community public services, effectively characterizing configuration characteristics. The Shannon-Wiener Diversity Index [42] was used to estimate community diversity. The higher the Shannon index, the greater the diversity. The core of facility aggregation is the degree of facility mixing, which is measured in a manner similar to land mixing. The Shannon-Wiener Diversity Index is widely used domestically and internationally to measure the spatial diversity characteristics of facilities.


$$H_{j}=-\text{Σ}P_{ij}\text{I}nP_{ij}\;\;\;\;\;\;\;\;\;\;\;\;\;\;\;\;\;\;\;\;\;\;\;\;\;\;\;\;\;\;\;\;\;\;\;\;\;\;\;\;\;\;\;\;\;\;\;\;\;\;\;\;\;\;\;\;\;\;\;\;\;\;\;\;\;\;\;\;\;\;\;\;\;\;\;\;\;\;$$ (1)

P_ij_ indicates the proportion of the i-th type of facility within the j-th unit relative to the total number of facilities in that unit, and H_j_ represents the diversity index of the various facilities within the j-th unit [39].

(2) Colocation quotient

Global Colocation Quotient (GCLQ) is a spatial statistic used to measure the spatial association between two or more categories of point observations and is widely applied in exploring spatial proximity across various scenarios [46,54]. GCLQ is defined as a probability ratio, where the denominator represents the expected proportion, and the numerator is the observed number of B-type elements neighboring A-type elements. GCLQ_A → B_ indicates the degree to which A is attracted to B; N represents the total number of points; N_A_ represents the number of A points; N_B_ represents the number of B points; N_A → B_ represents the number of A points that have B points as their nearest neighbors.


$$GCLQ_{A\to B}=\frac{N_{A\to B\;}/N_{A}}{N_{B}/\left(N-1\right)}\;$$ (2)

The GCLQ examines the existence of spatial association from the entire study area and the LCLQ further considers the spatial heterogeneity of clustering patterns [55]. LCLQ can be used to explore variations in the dependencies between a given class A-type and the surrounding subset of B-type points in different spaces. LCLQ combines global and spatial regression model geographic weighting parameters to indicate the likelihood of B-type elements appearing within the domain of A-type elements when each element of A-type is determined compared to the overall spatial distribution [47].

N_A_ and N_B_ represent the total number of elements A and B, respectively. N_A → B_ is the number of A nearest neighbor points to B. N is the total number of points in the study area. N_Ai → B_ is the weighted average of B in the neighborhood of A (Ai). f_ij_ is a binary variable, where f_ij_ = 1 if j is a B-type point; otherwise, f_ij_ =  0, and w_ij_ is the geographic weight indicating the importance of point j to point i.


$$LCLQ_{A\to B}=\frac{N_{Ai\to B\;}}{N_{B}/\left(N-1\right)}\;$$ (3)

$$N_{Ai\to B}=\Sigma_{\text{j}=1,（\text{j}\neq \text{i}）}^{N}\frac{W_{ij}gf_{ij}}{\Sigma_{\text{j}=1,（\text{j}\neq \text{i}）}^{N}W_{ij}}\;$$ (4)

A GCLQ_A → B_ and LCLQ_A → B_ value greater than 1 indicates that A is more likely to be attracted by B in the global [55] or local region, with higher values indicating stronger attraction; a value less than 1 indicates that A is more likely to be isolated from B in the global or local region; and a value of 1 indicates a tendency for random distribution between the two. Through the calculation of GCLQ and LCLQ, the spatial association of the entire region can be explored, local settlement patterns identified, and statistically significant hotspots located.

(3) Geographically weighted regression model

GWR is an improved spatial linear regression model based on spatially nonstationary data that reflects the heterogeneity of regression relationships through the variation in regression coefficients at different spatial locations. It provides local models of variables by fitting a regression equation for each feature [56]. This research overcomes the limitation of traditional regression models, which are unable to capture the variation in regression coefficients between facility colocation construction and social capital influenced by urban local environments.


$$y_{i}=\beta_{0}u_{i},v_{i}+\Sigma_{\text{i}=1}^{k}\beta_{k}u_{i},v_{i}x_{ik}+\theta_{i}\;$$ (5)

Where y_i_ is the observed value; (u_i_, v_i_) are the coordinates of the i-th sample point; β_0_(u_i_, v_i_) is the regression constant of the i-th sample point; β_k_(u_i_, v_i_) is the regression coefficient of variable k at the i-th sample point; k is the number of independent variables; x_ik_ is the value of independent variable x_k_ at point i; θ_i_ is the random error. The GWR module in ArcGIS Pro was used to construct the model.

### 3.3. Scope of research and data sources

**Fig 2:**
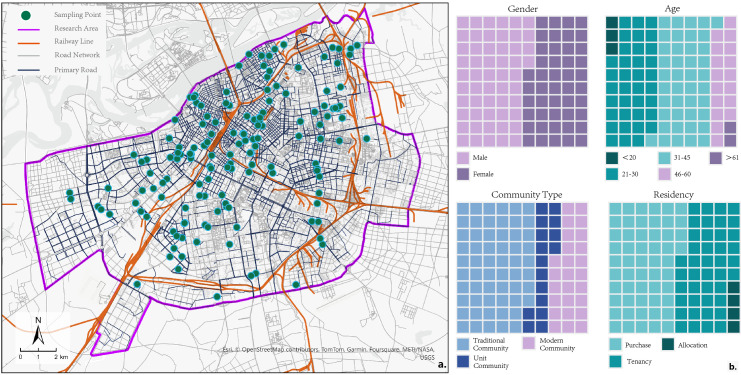
Summary of the scope of the study and research area (a) research area and sampling points (b) summary of gender, age, community type, and residency. Data source: ArcGIS Online basemap [57].

The area within the Third Ring Road of Harbin was selected as the research area (Fig 2a). As an urban core area, it has well-developed community public service facilities, such as healthcare, cultural education, and social security. It includes most typical communities, making it a suitable research area for this study. Information on community public service facility POIs in the main urban area of Harbin prior to December 2023 was obtained using the DaZhong Dian Ping API (https://www.dianping.com) (Table 1). This platform is one of the most widely used local life information platforms in China, offering a comprehensive POI database and detailed categorization. Referencing the “Standard for Urban Residential Area Planning and Design” (GB50180-2018), this study cleaned and classified the POI data, resulting in six main categories, 17 subcategories, and a total of 8,268 POI entries.

**Table 1:** Type and number of community public service facilities.

NO.	Main Categories	Number	Subcategories
1	Convenience Services Facilities	5201	Fitness Centers: 291, Financial Services: 1333, Water Delivery Stations: 179, Telecommunications Business Offices: 441, Logistics and Express Delivery: 1816, laundry: 1141 (6 categories)
2	Cultural and Sports Facilities	894	Community Activity Centers: 56, Community Cultural Centers: 68, Gymnasiums 770 (3 categories)
3	Administrative Services Facilities	479	Community public service: 473, Disability centers: 6 (2 categories)
4	Healthcare facilities	478	Community health service centers: 186, health service stations: 292 (2 categories)
5	Aged Care Facilities	163	Retirement facilities: 40, rehabilitation services: 123 (2 categories)
6	Childcare Facilities	1053	Kindergartens: 889, Childcare Centers: 164 (2 categories)
Total	Six categories	8268	17 categories

### 3.4. Characteristics of subjective survey samples

Data collection involved conducting surveys in typical communities from 20 August to 31 August 2024 using door-to-door questionnaires and random online surveys. A total of 198 questionnaires were distributed, and 164 valid questionnaires were returned. The locations of the sampling points are shown in Fig 2a. In the sample characteristics, most respondents were aged between 21 and 60, with a relatively balanced gender distribution. Community types mainly included traditional old neighborhoods, unit-assigned communities, and modern commercial communities, with similar proportions for each residential type (Fig 2b). The information obtained from the questionnaires included the social attributes of the residents and their scores on 14 questions related to social capital.

### 3.5. Variable description

Community public service facilities serve as interactive platforms, offering primary locations for residents to connect to their social networks. This study combines the existing Sense of Community Index [58] and social capital indicators to screen for potential issues related to the community environment, residents’ activity behaviors, and facility usage from dimensions such as network, local associations, sentiment, trust, solidarity, volunteerism, and cohesion.

With the trend of increased aging, the demand for community aged care [59] has increased, and the relaxation of the “second child” policy has led to a rise in newborns. The spiritual and cultural needs of residents are becoming increasingly diverse [60], and community social support must be more inclusive [61]. Therefore, users were segmented, and the indicators were broken down into three questions: Q6, Q7, and Q8. Based on the grassroots governance model [62] and the integration of diverse community entities, Q10 was summarized. Combining the Sense of Community Index [63], the original Q3 and Q13 of the social capital scale were made more specific and understandable (Table 2). The remaining semantically similar questions were merged to obtain 14 indicators. Respondents rated these items on a five-point Likert scale, where 1–5 represents “completely disagree” to “completely agree.”

**Table 2:** Research variables and measurement of social capital and community public service facilities.

Variables	NO.	Description of Indicators	Measurement Method
Social Capital of the Community	Q1	I am willing to spend some time or money on the renovation and development of the community.	Completely Disagree - Completely Agree = 1–5
	Q2	I often meet and greet with neighborhood residents.	
	Q3	I am willing to attend lectures, community groups, or interest workshops held nearby.	
	Q4	When I face issues, I frequently discuss and exchange views with my neighbors.	
	Q5	If I need to go on a long trip, I can rely on other community residents to help collect packages and mail.	
	Q6	I believe the neighborhood can adequately meet the needs of children.	
	Q7	I think older adults feel comfortable and convenient living in the community.	
	Q8	I think individuals with disabilities (visual, hearing, or mobility impairments) will receive assistance and support.	
	Q9	I feel that the overall environment of the neighborhood is quite suitable.	
	Q10	I have great trust and respect for the work of the neighborhood committee and property management.	
	Q11	I feel that the neighborhood is friendly, safe, and reliable.	
	Q12	I enjoy living in my current neighborhood.	
	Q13	Others easily recognize and remember my neighborhood or surroundings, such as nearby landmarks or buildings and representative community activities.	
	Q14	If someone organizes residents to solve neighborhood issues, I would actively participate.	
configuration characteristics		Shannon Diversity Index	$H_{j}$
		Proportion of Facility Spatial Units	*Pij*
Colocation pattern		Global and Local Colocation Quotients	*GCLQ, LCLQ*

**Fig 3:**
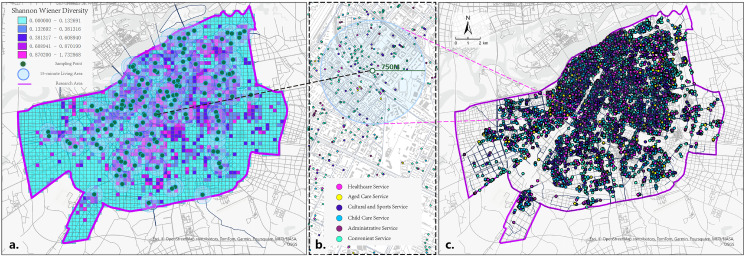
Facilities POI and diversity (a) diversity of facilities (b) extent of community life circle at sampling sites (c) distribution of POI. Data source: ArcGIS Online basemap [57].

In the supply configuration of the facilities, grid cells (300 m ×  300 m) were constructed based on a 5-minute walking range to calculate the Shannon-Wiener Diversity Index of six types of facilities within each grid cell, denoted as H_j_ (Fig 3a). This was used to explore the degree of aggregation of facility types within the smallest range. Based on the surveyed sampling points, buffers were constructed with an 800-meter radius (approximately 15 minutes walking distance), and the proportion of each of the six types of facilities within this unit range was calculated as P_ij_ (Fig 3b). GCLQ was calculated using the six types of facilities on a global scale, strong association patterns were filtered out with the GCLQ > 2, their LCLQ was calculated, and the strength of the attraction of the colocation pattern was assessed by the size of the LCLQ, which was then assigned to the sampling points within the unit range.

## 4. Results

### 4.1. Social capital of the community

We obtained subjective scores for 14 questions regarding community social capital through questionnaires and interviews, extracted indicators for the 14 dimensions, and verified their validity. Using SPSS 26.0, exploratory factor analysis was conducted on the indicators using principal component analysis. The KMO and Bartlett’s test results showed KMO =  0.859 > 0.5, P < 0.01, indicating strong correlations among the original variables; hence, they were suitable for factor analysis. The initial factor loadings were rotated using the orthogonal rotation method (Varimax) to clarify the structure of the factor-loading matrix. Four factors were extracted based on factor loadings > 0.5 and eigenvalues > 1, explaining 65.16% of the variance. By analyzing the factor loadings and combining them with the dimensions of social capital, four aggregate indicators were identified: **Cohesion** (Q1, Q2, Q3, Q4, Q5), **Solidarity** (Q6, Q7, Q8), **Community Trust** (Q9, Q10, Q11), and **Community Sentiment** (Q12, Q13, Q14) (Table 3). Regarding reliability, Cronbach’s alpha was used to test the internal consistency of the questionnaire data, with all values greater than 0.6 indicating strong internal data correlations for each indicator. **Cohesion** primarily includes social action networks and interactive activities; **Solidarity** represents home-based aged care, vulnerable groups, and an inclusive community environment; **Community Trust** includes trust in community organizations and the neighborhood environment; and **Community Sentiment** represents community identity, markers, and volunteering. According to the analysis of social capital characteristics, the surveyed residents had the highest sense of **Community Sentiment**, with more concentrated scores, and 59.4% of residents tended to actively solve community problems. However, **cohesion** was the lowest, with significant score fluctuations, and only 42% tended to participate in community organizations.

**Table 3:** Descriptive statistics of the data.

*Category*	*Items*	*Mean*	*Factor Loading*	*Reliability*	*Mean*	*SD*
***S1*** ***Cohesion***	***Q1***	***3.11***	***0.642***	***0.808***	***3.3***	***0.965***
	***Q2***	***3.57***	***0.661***			
	***Q3***	***2.95***	***0.776***			
	***Q4***	***3.36***	***0.607***			
	***Q5***	***3.36***	***0.686***			
***S2*** ***Solidarity***	***Q6***	***3.89***	***0.673***	***0.702***	***3.414***	***0.849***
	***Q7***	***3.45***	***0.684***			
	***Q8***	***3.08***	***0.632***			
***S3*** ***Trust***	***Q9***	***3.26***	***0.620***	***0.736***	***3.388***	***0.907***
	***Q10***	***3.2***	***0.704***			
	***Q11***	***3.43***	***0.756***			
***S4*** ***Sentiment***	***Q12***	***3.68***	***0.521***	***0.673***	***3.628***	***0.849***
	***Q13***	***3.8***	***0.833***			
	***Q14***	***3.69***	***0.539***			

### 4.2. Configuration characteristics of community public service facilities

The Shannon-Wiener Diversity Index (H_i_) was calculated within the grid cells of the study area and classified using the natural breaks method. As shown in the figure, the passage of trains caused a break in urban development, with the Shannon index values being higher in the south and East and lower in the north and West, using railway tracks as a boundary (Fig 3a). The diversity in facility construction in Harbin’s main urban area is significantly polarized, with a tendency toward single-type facility construction in many units.

**Fig 4:**
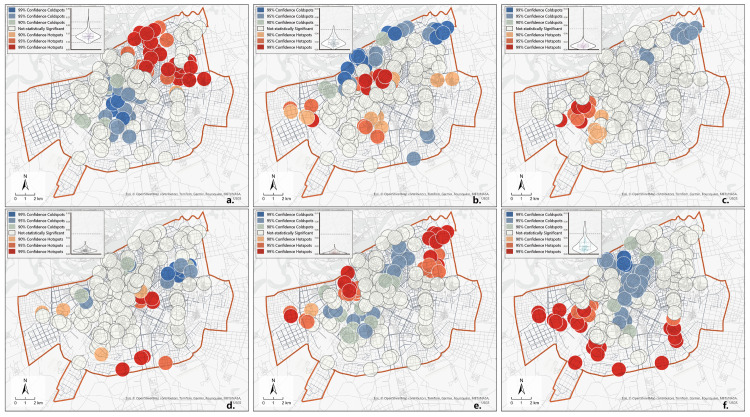
Hot spots and cold spots in terms of percentage of the number of facilities: (a) convenient service; (b) cultural and sports; (c) administrative; (d) healthcare; (e) aged care; (f) childcare. Data source: ArcGIS Online basemap [57].

Next, the proportion of each of the six types of community public service facilities within the unit space of the sampling points was calculated and denoted as P. Convenience service facilities had the most significant proportion, followed by childcare facilities. Aged care facilities had the lowest proportion, indicating that community aged care still needs to be strengthened (Fig 4). Finally, a hotspot analysis (Getis-Ord Gi * algorithm) [57] was conducted on the six P values to identify hot and cold spots of facility proportions and assess the spatial heterogeneity of these proportions. Finally, the P-values were analyzed using Getis-Ord Gi^ *^ to identify hot and cold spots of each facility’s proportion and evaluate the spatial heterogeneity of facility proportions. P-convenience services showed high-value clustering in the Northeast and low-value clustering in the Southwest of the study area, with the highest number of significant clusters (Fig 4a). P-cultural and sports and P-childcare facilities showed low-value clustering in the northern part of the city and high-value clustering in the southern part (Figs 4b and 4f). P-aged care facilities showed high-value clustering on the city outskirts and low-value clustering in the city center (Fig 4e). P-administrative and P-medical facilities were clustered in the south and dispersed in the north, with a few significant points indicating a uniform distribution (Figs 4c and 4d).

### 4.3. Colocation patterns of community public service facilities

**Fig 5:**
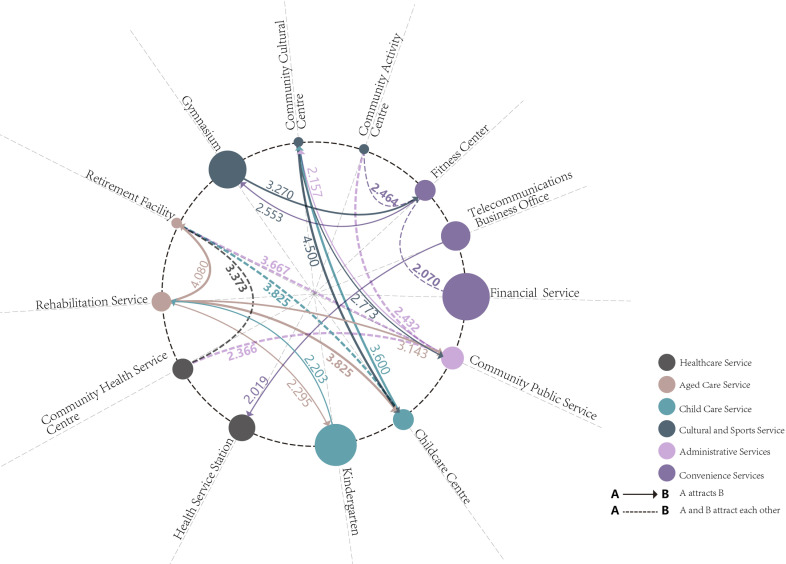
GCLQ of community public service facilities.

First, the colocation patterns of facilities across the entire area were calculated using the GCLQ to obtain the overall association patterns, followed by the LCLQ to further determine the distribution characteristics of the significant associations. In accordance with previous research, the 10-order neighbor was used as the adaptive bandwidth, which is most suitable for spatial association at the study area scale [53]. When the GCLQ is close to 1, it represents a significantly dispersed pattern among the facilities. This study selected colocation patterns with a GCLQ >  2 and P <  0.05, representing strong significant associations [54]. A total of 24 association patterns involving 13 facilities across the entire area were identified (Fig 5). The larger the circle, the greater the total number of facilities; dashed lines indicate mutual attraction and solid lines indicate one-way attraction (i.e., A → B means A attracts B); the thicker the association line, the stronger the association. The four strongest colocation patterns were Community Cultural Services →  Childcare Center (4.500), Rehabilitation →  Retirement Facility (4.080), Childcare Center →  Retirement Facility (3.825), and Rehabilitation →  Childcare Center (3.825). The overall attractiveness of aged care, cultural and sports, and childcare facilities is relatively strong. The overall association patterns were as follows:

1. Mutual attraction colocation patterns exist among facilities such as retirement facilities, community public service facilities, retirement facilities and childcare centers, childcare centers, and community cultural service facilities.
2. Facilities within the same main category have associations such as rehabilitation services →  retirement facilities, fitness centers →  financial services, indicating that facilities with similar functions tend to attract each other.
3. Facilities with overlapping user groups are associated, such as retirement facilities →  community health service centers, and community cultural services →  childcare centers. These associations provide multiple services to both older adults and their children.

**Fig 6:**
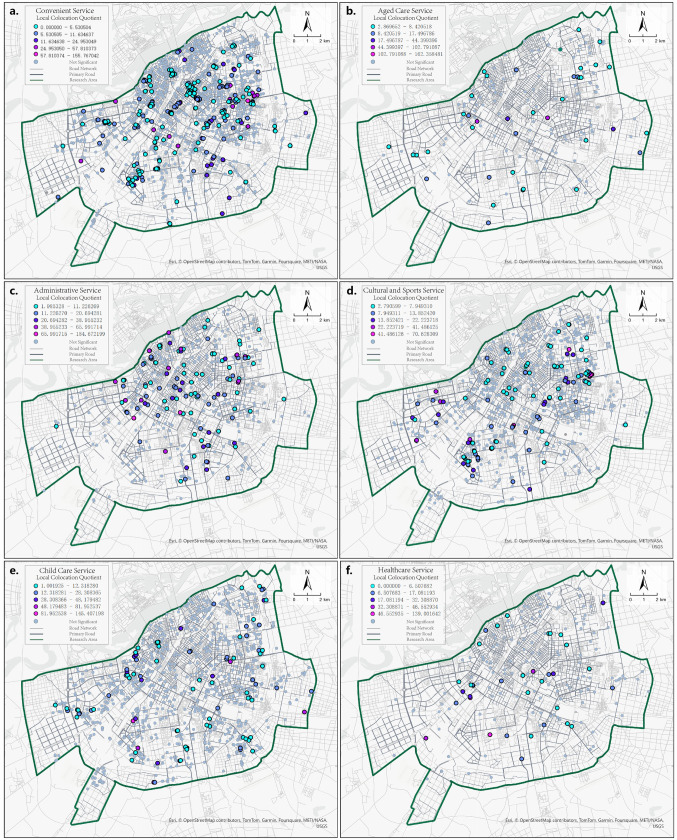
LCLQ of community public service facilities. Data source: ArcGIS Online basemap [57].

In the second step, based on the 24 strong colocation patterns identified by GCLQ, the LCLQ values for the six types of facilities and their significantly associated facilities were calculated to detect the colocation patterns and degrees within their local ranges (Fig 6). To explore the correlation patterns of community public service facilities within the smallest community activity range, a bandwidth of 300 meters (five-minute living circle) was set, and statistical significance was tested. Integrating all colocation patterns with GCLQ > 2 within the same subcategories (e.g., Fig 6a shows all association points related to convenience services, such as gymnasiums, community activity centers, and financial services being attracted by fitness centers; fitness centers being attracted by financial service facilities, and community health service centers being attracted by telecommunications business offices. These association points are represented by LCLQ convenience, indicating the LCLQ of convenience service facilities and their associated facilities. (The same principle is applied to the remaining five categories.) As the calculation filters strong association rules, all six types of facilities show significant colocation clustering patterns, with only a few isolated points (9) in convenience service facilities. Surprisingly, the probability of high LCLQ association points appearing in the urban core area was lower than that in the new urban or peripheral areas, indicating that facilities in the core area tended to be more homogeneously matched. In contrast, in peripheral or new urban areas, facilities tended to form a clustered construction model.

LCLQ-convenience association points show characteristics of large quantity, high degree, and uniform distribution, indicating a strong synergy in the city’s overall facility coordination. LCLQ-cultural and sports and LCLQ-administrative association points were numerous and evenly distributed. The former’s association points are primarily located near new urban areas and university towns, with lower averages, whereas the association points of administrative service facilities have no significant locational characteristics. LCLQ-childcare, LCLQ-healthcare, and LCLQ-aged-care association points were relatively unevenly distributed and fewer in number but had higher average LCLQ values. This indicates that these types of facilities are highly attractive; however, the fairness of resource allocation must be improved across the entire area.

### 4.4. Association between community public service facilities and social capital

**Fig 7:**
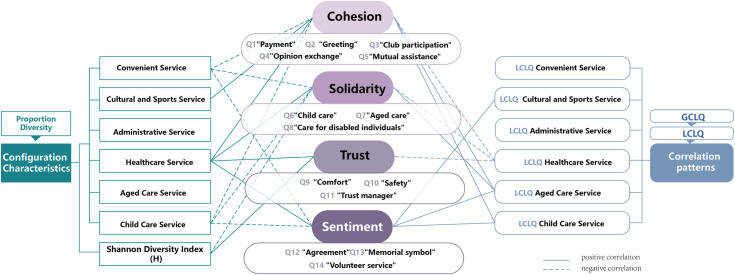
Overview of the OLS model.

To further statistically analyze the relationship between facility association patterns and community social capital, an 800-meter radius was set as the buffer zone, obtaining the LCLQ values of facilities around the survey sampling points, representing the strength of colocation patterns of facilities within the daily living range of the surveyed subjects. This study aimed to assess the impact of the association patterns of facilities on community social capital. Hence, isolated points were not considered, and if there were multiple values of the same association within the sampling point range, the average value was taken. Statistical analyses were conducted to understand the impact mechanisms of different association strengths on community social capital. The independent variables were normalized using Z-scores in SPSS, followed by OLS model validation, which showed no multicollinearity among the factors. The configuration characteristics and colocation patterns of community public service facilities are associated with the mechanisms of community social capital (Fig 7).

The results indicate that more facility construction is not always better; instead, mutual coordination and joint development are required. The diversity of facility types has a positive impact on community trust but a negative impact on solidarity. Urban areas with more types of facilities may have high vitality and complex travel conditions that are detrimental to the lives of vulnerable groups. Based on facility configuration characteristics and colocation patterns, the following rules were observed:

1. P-convenience was negatively correlated with community cohesion, solidarity, and sentiment in social capital, with no significant impact on community trust. Although LCLQ convenience was not significantly associated with social capital, it had no negative effects. Existing studies have found that high-density or excessive commercial land use adversely affects community capital, and the more facilities there are for daily needs, the lower the residents’ local identity. However, the joint construction of convenience and related facilities helps eliminate these negative feelings and can serve as a reference for composite construction.
2. Although P-aged care was not significantly associated with social capital, P-child care was negatively correlated with community cohesion and sentiment in social capital. However, LCLQ-aged care and LCLQ-childcare had significant positive effects on community cohesion, solidarity, and sentiment. The spontaneous construction of these two types of facilities positively affects social capital and can serve as a reference for subsequent composite construction.
3. P-healthcare was positively correlated with social capital in all four dimensions, and healthcare facilities had a strong impact on its enhancement. However, the colocation patterns of healthcare facilities with aged care and community public service facilities adversely affected social capital. Considering the trend of integrated medical and aged care, this may be due to existing imperfect configurations or conflicts with municipal medical resources, resulting in low social capital.
4. P-cultural and sports and LCLQ-cultural and sports had significant positive impacts on community cohesion and sentiment. The construction of facilities, such as sports venues and community cultural centers, can significantly enhance community social capital. P-administrative and LCLQ-administrative had no significant overall impact on social capital, possibly because of issues such as the small scale and low utilization of this type of facility construction (Table 4).

**Table 4:** Parameter estimation and test results of the OLS model.

Variables	S1 Cohesion		S2 Solidarity		S3 Trust		S4 Sentiment		VIF
	Beta	S.E.	Beta	S.E.	Beta	S.E.	Beta	S.E.	
Intercept	3.221***	0.055	3.253**	0.054	3.302**	0.067	3.720**	0.054	
P-convenience	−0.328**	0.058	−0.240***	0.057	0.025	0.086	−0.172***	0.060	1.462
P-cultural and sports	0.127 *	0.075	0.059	0.060	−0.080	0.090	0.050	0.070	1.322
P-administrative	0.002	0.056	0.030	0.045	0.088	0.107	0.062	0.055	1.179
P-healthcare	0.141***	0.061	0.258***	0.064	0.356***	0.085	0.235***	0.072	1.186
P-aged care	0.110	0.064	0.095	0.069	0.091	0.083	0.044	0.070	1.491
P-childcare	−0.277***	0.099	0.253	0.094	−0.139	0.095	−0.295**	0.088	1.849
H	−0.113	0.066	−0.171**	0.079	0.235**	0.095	−0.069	0.066	1.942
LCLQ-convenience	0.047	0.091	−0.045	0.081	0.020	0.122	0.012	0.076	1.555
LCLQ-cultural and sports	0.039	0.131	0.085	0.097	0.051	0.135	0.152 *	0.105	2.159
LCLQ-administrative	0.022	0.061	0.001	0.053	-0.015	0.079	-0.027	0.050	1.306
LCLQ-healthcare	-0.217***	0.041	-0.055**	0.027	-0.249**	0.066	-0.093	0.061	1.177
LCLQ-aged care	0.299***	0.038	0.195***	0.043	-0.089	0.064	0.280***	0.037	1.222
LCLQ-childcare	0.108 *	0.062	0.035	0.043	0.029	0.070	0.133**	0.044	1.329
Adjusted R^2^	0.252		0.163		0.128		0.211		
AICc	386.013		375.635		448.323		364.181		

*^ ,^ *^*^, and ***indicate significance at α = 0.1, α = 0.05, and α = 0.01, respectively.

**Fig 8:**
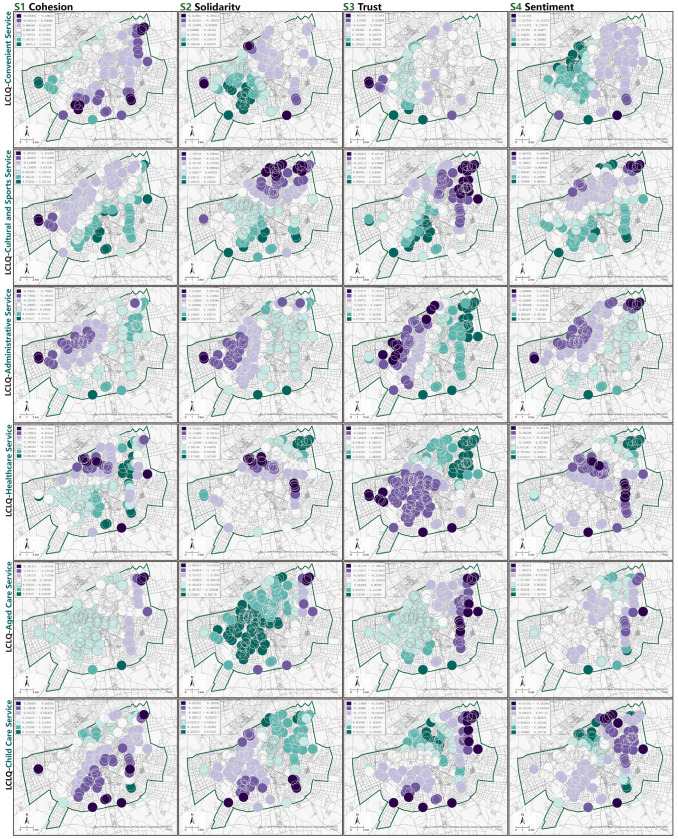
The GWR coefficients of GCLQ factors for community public service facilities. Data source: ArcGIS Online basemap [57].

The traditional linear regression model provides a global estimate of the parameters, and the resulting coefficients of the independent variables are homogenized because of the nature of the global model [40]. To address the inseparable colocation phenomenon associated with geographic factors, a GWR model was used to explore the spatial heterogeneity of the impact of facility aggregation on community social capital, reflecting the variation in regression coefficients across different urban areas. Before constructing the GWR model, it is necessary to exclude the influence of multicollinearity among factors by removing variables with a Variance Inflation Factor (VIF) greater than 7.5 in the OLS model. Calculations revealed that all VIF values in the OLS equation were significantly below 7.5, with the GWR model exhibiting a higher Adjusted R^2^ and lower corrected Akaike information criterion (AICc) than the OLS model, indicating that GWR provides better explanatory results than the OLS model (Table 5). Each spatial unit in the GWR model has specific coefficients, and the average and median values of these coefficients are calculated to provide an overall assessment. The standardized residual values of the GWR were subjected to Global Moran’s I calculation, revealing that none of the four model sets exhibited spatial autocorrelation (−1.65 < Z < 1.65, P > 0.1) and were randomly distributed in space, thus proving that the overall effect of the model is interpretable. Given that this study further investigates the spatial heterogeneity of the impact of facility connectivity on social capital by examining whether it is influenced by the urban environmental context, it aims to guide integrated facility construction. Fig 8 illustrates the spatial variation of the coefficients of the impact of facility colocation patterns on community social capital.

**Table 5:** Parameter estimation and test results of the GWR model.

		S1 Cohesion		S2 Solidarity		S3 Trust		S4 Sentiment	
		Median	Mean	Median	Mean	Median	Mean	Median	Mean
Factor coefficients	LCLQ-convenience	0.098	0.161	0.097	0.194	0.109	0.129	0.169	0.254
	LCLQ-cultural and sports	−0.010	−0.011	−0.123	−0.217	−0.064	−0.119	0.038	0.031
	LCLQ-administrative	−0.072	−0.053	0.056	0.016	0.027	0.090	−0.076	−0.083
	LCLQ-healthcare	−0.205	−0.249	−0.053	−0.005	−0.036	0.257	−0.119	−0.056
	LCLQ-aged care	0.338	0.147	0.172	−0.213	−0.062	−0.384	0.405	0.443
	LCLQ-childcare	0.079	0.079	0.027	0.081	−0.006	0.025	0.037	0.019
Model Indicators	R^2^	0.753		0.767		0.752		0.746	
	Adjusted R^2^	0.655		0.674		0.654		0.6443	
	AICc	295.108		257.276		333.311		270.001	
	Moran’s I	0.053		0.009		-0.014		−0.009	
	Z	1.10		0.516		-0.266		−0.104	

1. The association construction of medical and health facilities with aged care services and community public service facilities generally had a negative impact on community cohesion across the entire area (Fig 8-A4). In terms of social support, community sense of belonging, and community trust, the correlation coefficient of the LCLQ medical decreased and became negative from the Northeast to the Southwest (Figs 8-B4, 8-C4, and 8-D4). In terms of the effects on Solidarity, Sentiment, and community trust, the correlation coefficients of LCLQ-healthcare gradually decreased from Northeast to Southwest and became negative (Figs 8-B4, 8-C4, and 8-D4). According to the urban layout, the linkage construction of healthcare facilities primarily shows significant negative correlations in commercial districts such as Zuo Zhan and convention centers.
2. LCLQ-aged care shows a partial negative correlation with community social capital on the eastern side of the study area, gradually turning positive and increasing in impact on the western side (Figs 8-A5, 8-B5, and 8-D5). The joint construction of aged care facilities with rehabilitation therapy facilities, kindergartens, and community service centers in new urban areas and school cluster zones can enhance community cohesion, support, and sentiment. However, this did not enhance the community’s perception of trust (Fig 8-C5).
3. According to the regression coefficient statistics, the LCLQ-childcare has a largely positive impact on community social capital, with the proportion of positive coefficients affecting community cohesion reaching 84.15%. In contrast to LCLQ-healthcare, joint construction of kindergartens, daycare centers with aged care facilities, and community cultural service facilities in central and northern urban areas—core residential and commercial zones—effectively promotes social capital. Additionally, negative correlations with community trust and sentiment in the northeastern areas may be indirectly related to urban security in the Daoli district (Figs 8-A6, 8-B6, 8-C6, and 8-D6).
4. Regarding the association between LCLQ-cultural and sports, and community sentiment, 72.06% of the regression coefficients were positive. The regression coefficients of LCLQ culture showed a gradual decrease from south to north in the study area (Figs 8-A2, 8-B2, 8-C2, and 8-D2). The southern region, with its numerous universities and primary schools, has a concentrated layout of facilities related to culture and entertainment. Overall, this layout positively affected community social capital. In contrast, mismatched functional focus in the northern urban area showed a negative correlation.
5. The LCLQ-convenience index was not significant in OLS. However, in the GWR model, approximately 50% of the sampled points in the newly developed northwest urban areas showed a significant positive correlation with sentiment (Fig 8-D1). Therefore, joint construction of supermarkets, logistics facilities, and fitness facilities should be considered in similar regions. In the GWR model of solidarity, LCLQ-administrative services showed significant positive correlations in the southeast urban areas, accounting for approximately half of the total samples (Fig 8-C3). This indicates that the joint construction of community public service facilities in old city areas positively supports community life.

In exploring the mechanisms affecting social capital, the regression coefficients of LCLQ-aged care were generally higher in the West than in the East. Simultaneously, LCLQ-healthcare and LCLQ-administrative services were higher in the Northeast than in the Southwest. The regression coefficients of LCLQ-childcare and LCLQ-culture and sports were higher in the south than in the north. Moreover, LCLQ-childcare was positively correlated with popular business districts, whereas LCLQ-healthcare showed a negative correlation. LCLQ culture and sports, as well as LCLQ-aged care, were positively correlated with universities and research institutes.

## 5. Discussion

### 5.1. Colocation patterns of community public service facilities and their influencing factors

The study established a facility association network aligned with common sense and previous research [64], showing that while similar facilities tend to cluster, elderly care facilities are the most appealing, followed by community cultural centers and nurseries. These findings emphasize the daily entertainment and healthcare needs of older adults and young children, thus addressing the second research question. The study’s emphasis on the importance of an intergenerational integrated facility construction model aligns with related research perspectives [65]. Facilities integrating medical care with elderly care and combining elderly and childcare services conform to residents’ natural living patterns and should be considered a top priority in urban renewal efforts.

Unexpectedly, in the colocation quotient calculations in the “configuration characteristics of community public service facilities” section of this paper, the probability of high LCLQ-childcare values is lower in urban core areas than in newly developed or border areas. Existing research on the colocation patterns of medical facilities has found that basic (local services) and specialized (city-level services) medical facilities, while somewhat related, are spatially mutually exclusive [51]. This study provides a partial explanation for this result. A potential explanation for this phenomenon is the limited total number of facilities in non-core or peripheral areas, where the population of newly settled young and middle-aged residents at the urban expansion boundary exhibits a high demand for childcare and medical services. Another possibility is that facilities within urban areas have high stock and density, and other facilities may have mutually exclusive or incompatible effects on them, resulting in LCLQ values not significantly increasing in high-density mature areas.

### 5.2. Impact of facility-related construction on social capital

This study found that not all facilities or associations had positive effects on community capital. Unlike previous research [7], the diversity of facility construction can enhance community trust, although it has a negative impact on community solidarity. The data on P-convenience and LCLQ-convenience show that excessive construction of convenience service facilities is not conducive to promoting community social capital and does not inspire residents’ place identity. This is consistent with research results on the association between community facilities and sense of place [11]. In addition, a joint layout of convenience, childcare, and aged care facilities with related facilities is more conducive to increasing social capital than simply increasing the number of such facilities. Therefore, such self-organized colocation patterns enhance residents’ user experience and have promotional value, thus addressing the third research question.

This study primarily explores the relationship between facility construction and social capital using regression models, which are constrained by certain simplifications. Future improvements could involve more complex functional forms or other machine learning methods. Meanwhile, the research findings may also be influenced by other factors, such as regional culture, population density, and site density, necessitating further exploration of these differences across various environments in future studies.

### 5.3. Insights for urban supply characteristics and integrated facility planning

The study can serve as a foundational information collection method for planning community public service composite facilities, identifying social needs from the bottom up, and providing a reference for urban facility construction proposals. Policymakers should avoid viewing facility construction solely from a functional perspective and instead adopt an integrated and holistic approach to enhancing social capital [11]. Based on the spatial heterogeneity of the development of urban areas, i.e., the development objectives and positioning of different regions and the shortcomings of social capital, it is possible to specify a more compatible pattern of synergistic construction of facilities in different regions, which can lead to a distribution of features that are harmonized with the urban pattern [56].

Regarding the differentiated impacts on social capital across Harbin’s urban development zones, the city can be categorized into traditional old districts (eastern and northern parts), core commercial zones (northern parts), core educational and cultural zones (central and southern parts), and new urban zones (southwest parts). The old districts have relatively complete but aging infrastructure, and enhancing the construction of community medical centers and integrated service centers can improve community trust. The core commercial zones are densely populated and highly vibrant, making them suitable for building children’s interest classes and activity care centers to support intergenerational activities. Core educational and cultural zones, which are dominated by higher education institutions, can significantly enhance various dimensions of social capital through the joint construction of elderly universities and cultural activity centers. Urban new zones, located on the periphery and relatively underdeveloped, should prioritize basic needs by constructing comprehensive supermarkets and convenience service stations.

However, this study primarily examines the attraction of one type of facility to other facility types, lacking broader consideration of other environmental factors within the region. In future studies, a more comprehensive and holistic depiction of the interconnections and impacts within community life circles is desired. In this study, Euclidean distance was used to determine the neighborhood range; however, incorporating factors such as travel distance and time costs would provide a more accurate evaluation of facility construction associations. The next step could involve constructing urban networks to enable more refined and in-depth research.

## 6. Conclusions

This study proposes dimensions and indicators of community social capital that are more suitable for development needs, calculates the configuration characteristics of community public service facilities, explores the colocation patterns of facilities, and constructs OLS and GWR models. This confirms that the related construction of community public service facilities has a significant impact on the stock of community social capital and explores the spatial heterogeneity of this impact. The main conclusions are as follows:

1. Based on existing social capital research, this study incorporates contemporary needs by adding indicators for home-based aged care, grassroots governance, and inclusive environments. Through reliability and validity analyses, a community social capital index scale was developed, consisting of four dimensions: community cohesion, solidarity, community trust, and sentiment.
2. GCLQ calculations identified 24 significant associations among facilities, with the strongest four being: community cultural service facilities →  childcare centers (4.500), rehabilitation services →  retirement facilities (4.080), childcare centers →  retirement facilities (3.825), and rehabilitation services →  childcare centers (3.825). Locally, convenience facilities have the most evenly distributed association points, followed by cultural and sports, administrative, aged care, childcare, and healthcare facilities, which have fewer and unevenly distributed association points.
3. The OLS model shows that P-convenience and P-childcare are negatively correlated with community social capital, and P-aged care was not significantly correlated with community social capital. In contrast, LCLQ-aged care and LCLQ-childcare were positively correlated, while LCLQ-convenience was not significantly correlated with community social capital. This indicates that the joint construction of aged-related and childcare-related facilities can effectively enhance community cohesion, solidarity, and sentiment. The joint construction of convenience facilities may alleviate the inconvenience caused by the over proliferation of convenience facilities encroaching on other types of facilities. Although P-healthcare was positively correlated with community social capital, LCLQ-healthcare had a negative impact on social capital, and its related construction model still needs to be explored.
4. In exploring the mechanisms affecting social capital, the coefficients of the regression model summarized based on the GWR model were as follows: LCLQ-aged care was greater in the West than in the East; LCLQ-healthcare and LCLQ-administrative were greater in the Northeast than in the Southwest; and LCLQ-childcare and LCLQ-cultural and sports were greater in the south than in the north. Additionally, LCLQ-childcare was positively correlated with popular commercial areas, whereas LCLQ-healthcare was negatively correlated with them. LCLQ culture and sports are positively correlated with universities and research institutes. The colocation pattern of facility layout and its association with social capital are significantly influenced by the urban regional location and primary functional areas.

## Supporting information

S1 Final Data Community public service facilities POI and social capital. (XLSX)
